# Rare Gram-Negative Spinal Epidural Abscess Due to Klebsiella pneumoniae Bacteremia: A Case Report

**DOI:** 10.7759/cureus.101125

**Published:** 2026-01-08

**Authors:** Anais Panossian, Timmer Verhaegh, Aram A Namavar

**Affiliations:** 1 Internal Medicine, University of California, Los Angeles, USA; 2 Medicine, University of California, Los Angeles, USA; 3 Medicine, University of California, Los Angeles (UCLA) Health, Los Angeles, USA

**Keywords:** gram-negative bacteremia, klebsiella pneumoniae, lumbar spine infection, spinal epidural abscess, vertebral fracture

## Abstract

Spinal epidural abscesses (SEA) are rare but potentially devastating infections with high morbidity and mortality. Most often, a SEA is caused by *Staphylococcus aureus* and, on rare occasions, by Gram-negative bacteria such as *Klebsiella pneumoniae*. The incidence of SEA appears to be rising due to increased spinal instrumentation and enhanced detection with magnetic resonance imaging (MRI). In this case report, we discuss a case of a 74-year-old male patient with *K. pneumoniae* bacteremia secondary to a urinary tract source, complicated by lumbar SEA, L4-L5 osteomyelitis, and vertebral fracture requiring urgent decompression. The patient was successfully treated with surgical drainage and six weeks of intravenous ceftriaxone. This case highlights the importance of maintaining a high index of suspicion for rare causative organisms of SEA in patients presenting with systemic infection and back pain.

## Introduction

A spinal epidural abscess (SEA) is a rare but potentially devastating infection characterized by infection in the epidural space, often leading to spinal cord compression and irreversible neurological deficits if not promptly diagnosed and treated [1]. Risk factors that can predispose one to the development of a SEA include being immunocompromised, intravenous (IV) drug use, direct instrumentation to the spine, and bacteremia [2]. Clinically, a SEA can present with fever, back pain, and neurological deficits, though this classic triad is only common in 8%-15% cases of SEA [1]. Timely evaluation and treatment when there is clinical suspicion is critical in preventing serious complications and is therefore an important predictor of outcome [2]. Historically, SEA was thought to require urgent surgical decompression to avoid permanent neurological deficits or death. However, with the development of more advanced diagnostic tools such as magnetic resonance imaging (MRI), SEA is now being diagnosed earlier in the course of disease and has successful outcomes with medical management [3].

The most common pathogen known to cause a SEA is *Staphylococcus aureus*, accounting for approximately 60%-70% of cases. *Streptococcus* is an uncommon cause of epidural abscess, which can be a complication of vertebral osteomyelitis [4]. Gram-negative organisms, including *Escherichia coli* and *Pseudomonas aeruginosa*, are far less frequent, and *Klebsiella pneumoniae* is considered an exceedingly rare cause of SEA (~1%) [5]. These bacteria can enter the epidural space through direct inoculation, hematogenous spread, or extension from nearby infected tissues [1]. Here, we present a case of *Klebsiella* bacteremia causing SEA with further complications requiring a combination of surgical intervention and medical management.

## Case presentation

A 74-year-old male patient with a history of hypertension, diastolic heart failure, and nephrolithiasis was initially admitted on December 31, 2024, for an exacerbation of heart failure with preserved ejection fraction (HFpEF) and subacute painful leg swelling found to be cellulitis. During his admission, he was found to have a large left renal stone obstructing the ureter. Urology was consulted and placed a ureteral stent on January 3, 2025, with a plan for definitive stone treatment, outpatient 2-3 weeks post discharge.

**Figure 1:**
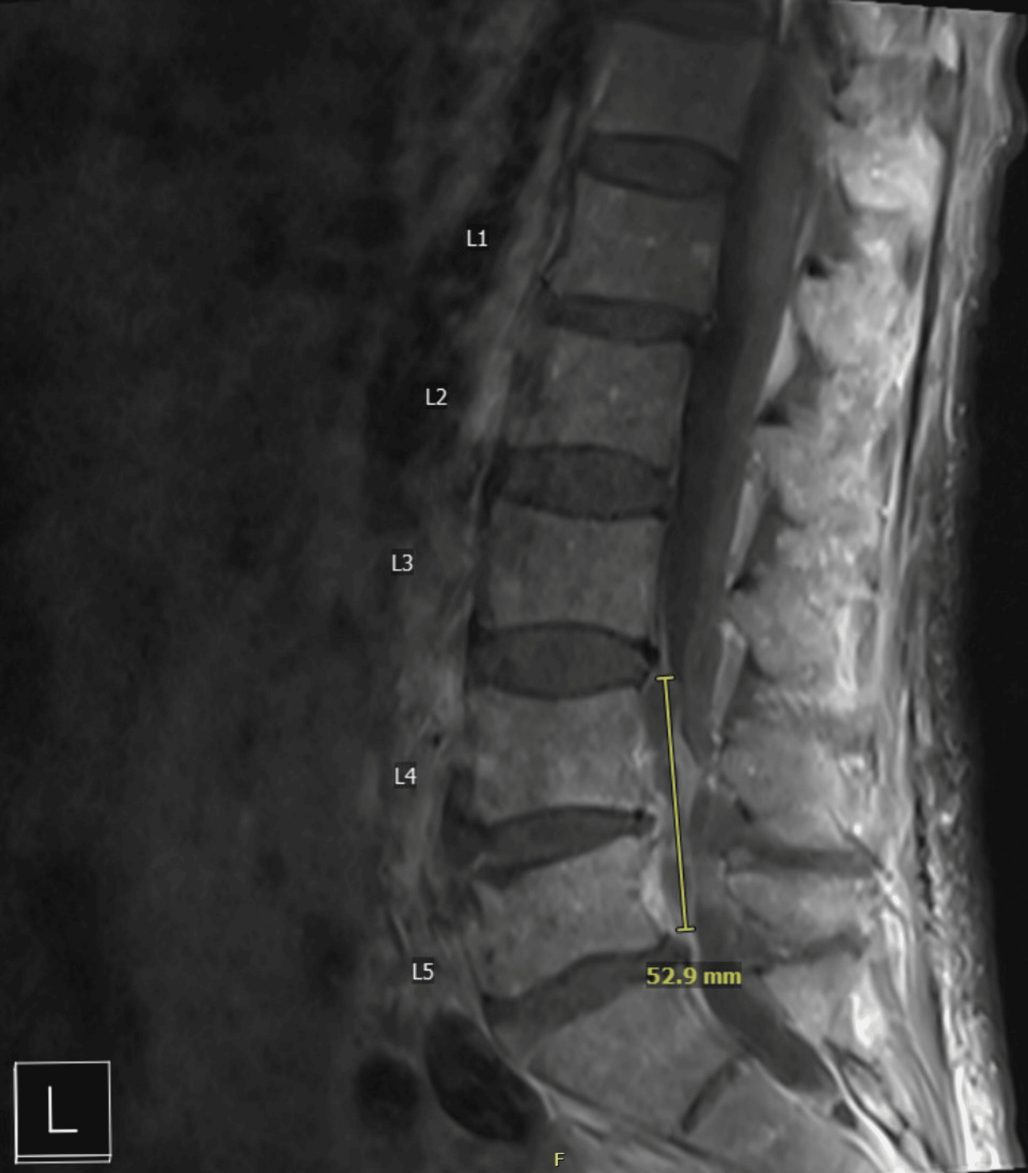
Sagittal magnetic resonance imaging (MRI) of the lumbar spine demonstrating a localized paraspinal abscess/phlegmon at the L4–L5 level. The yellow line delineates the craniocaudal extent of the abscess, measuring approximately 52.9 mm.

The patient’s hospital course was complicated by *K. pneumoniae* bacteremia, suspected to have originated from an obstructing ureteral stone. The patient subsequently developed acutely worsening lower back pain, worsening lower extremity pain and weakness, and new fecal incontinence. On physical exam, he had spinal tenderness to palpation over the L4-L5 region as well as L4-L5 radicular pain and paresthesias. MRI of the lumbar spine demonstrated a loculated epidural abscess/phlegmon at L4-L5 with severe spinal canal stenosis, vertebral osteomyelitis, and an L5 vertebral body fracture (Figure 1). Orthopedic surgery was consulted, and the patient underwent urgent decompressive laminectomy and drainage of the abscesses on January 14, 2025. Intraoperative cultures obtained on January 14 grew *K. pneumoniae*, consistent with the organism isolated from prior urologic cultures. Infectious disease was consulted, and the patient was initiated on IV ceftriaxone (2 g every 24 hours), with a planned treatment duration of six weeks, after which he recovered with no further complications.

## Discussion

This case contributes to a growing but still limited body of literature documenting *K. pneumoniae* as an uncommon but clinically significant causative agent of SEA - a life-threatening condition if not diagnosed promptly and treated appropriately [5]. The pathophysiology of SEA typically involves microbial seeding of the epidural space, most commonly via hematogenous dissemination [1,2,6]. Once seeded, bacterial proliferation triggers local inflammation, tissue necrosis, and abscess formation, which may result in spinal cord or nerve root compression and irreversible neurologic injury if treatment is delayed [6]. Various risk factors including diabetes, being immunocompromised, and direct spinal instrumentation can lead to the development of SEA; however, hematogenous spread from distant sites including urinary and respiratory tract or skin and soft tissue infection constitutes nearly half of SEA cases [3]. The lumbar spine is the most common site for a SEA (48%), followed by thoracic (31%) and cervical (24%) regions [7]. The clinical presentation of a SEA is often nonspecific, and the classic triad of fever, back pain, and neurological deficit is present in only 8%-15% of cases [1,6]. The diagnostic ambiguity highlights the importance of maintaining a high index of suspicion in patients with bacteremia - especially Gram-negative bacteremia - who develop new or progressive back pain, even in the absence of overt neurologic findings [6,8]. In our patient, early recognition was facilitated by documented bacteremia and subsequent neurologic deterioration, prompting timely imaging and intervention.

The cornerstone of diagnosis and management in vertebral osteomyelitis is microbiologic confirmation. While blood cultures may identify the causative organism in a subset of patients, image-guided biopsy or intraoperative tissue sampling is crucial, particularly when blood cultures are negative or atypical organisms are suspected [8]. In our case, operative cultures guided definitive antibiotic selection, reinforcing the importance of tissue diagnosis in optimizing outcomes and minimizing unnecessary broad-spectrum antibiotic exposure.

Prior reports of *K. pneumoniae* SEA describe a wide spectrum of presentations and management strategies. For example, an older woman with bacteremia but no neurologic deficit was treated conservatively with IV antibiotics and recovered [9]. By contrast, there have been several reported cases of SEA with neurologic compromise or extensive disease that have required surgery: a full-axial, community-acquired extended spectrum beta-lactamase (ESBL)-producing *K. pneumoniae* SEA (with source traced to pyelonephritis) in a diabetic patient that required multilevel decompression [5], cervical SEA managed with decompressive corpectomy and fusion with good recovery [10], and a case of SEA with cauda equina syndrome improved after delayed (>48 hours) decompression plus antibiotics [11]. Compared to these cases, our patient had bacteremia from a suspected urologic source, vertebral osteomyelitis with fracture, and neurologic compromise, aligning more with reports favoring early surgical decompression plus culture-directed therapy when neurological compromise is present, differing from the conservatively managed, neurologically intact case. Together, these data highlight that the pathogenesis of *K. pneumoniae* SEA likely involves hematogenous dissemination from a distant infectious focus such as the urinary tract, is frequently associated with diabetes, and requires individualized management based on neurologic status and extent of infection.

Given the increasing prevalence of multidrug-resistant *K. pneumoniae* strains, early recognition and targeted antimicrobial therapy are critical. In this case, timely surgical intervention and antibiotic initiation based on susceptibility profiles allowed for the successful recovery of the patient and highlight the importance of culture-guided treatment. It is important to note the role of multidisciplinary involvement - including infectious disease, orthopedic surgery, and urology - in the optimization of outcomes. Expanding documentation of such cases will help clarify prognostic factors and inform therapeutic guidelines for rare Gram-negative spinal infections.

## Conclusions

In conclusion, SEA is a rare but serious infection. This case highlights the importance of considering atypical Gram-negative pathogens such as *K. pneumoniae* in the differential diagnosis of SEA, especially in patients with comorbidities known to increase the risk of occurrence of a SEA. Further, this case highlights how heightened clinical awareness of these rare presentations, prompt imaging, and culture-guided antibiotic therapy are critical to favorable outcomes. Our patient’s successful recovery demonstrates that timely decompression combined with culture-guided antibiotic therapy can lead to favorable neurological and functional recovery. More identification and reporting of such cases are needed to guide optimal management strategies for this serious condition.

## References

[1] Sharfman ZT, Gelfand Y, Shah P (2020). Spinal epidural abscess: a review of presentation, management, and medicolegal implications. Asian Spine J.

[2] Hall WA, Munakomi S, Mesfin FB (2025). Spinal epidural abscess. StatPearls [Internet].

[3] Arko L 4th, Quach E, Nguyen V, Chang D, Sukul V, Kim BS (2014). Medical and surgical management of spinal epidural abscess: a systematic review. Neurosurg Focus.

[4] Chang CY (2023). Vertebral osteomyelitis and epidural abscess due to Group C and G Streptococcus. Arab Board Med J.

[5] Dang V, Rajkumar A (2018). Spinal epidural abscess caused by a community acquired extended spectrum beta lactamase producing Klebsiella pneumonia. IDCases.

[6] Darouiche RO (2006). Spinal epidural abscess. N Engl J Med.

[7] Sendi P, Bregenzer T, Zimmerli W (2008). Spinal epidural abscess in clinical practice. QJM.

[8] Berbari EF, Kanj SS, Kowalski TJ (2015). 2015 Infectious Diseases Society of America (IDSA) clinical practice guidelines for the diagnosis and treatment of native vertebral osteomyelitis in adults. Clin Infect Dis.

[9] Araújo F, Ribeiro C, Silva I, Nero P, Branco JC (2012). Klebsiella pneumoniae spinal epidural abscess treated conservatively: case report and review. Acta Reumatol Port.

[10] Kim MS, Cho DC, Sung JK (2008). Spontaneous cervical spondylodiscitis and epidural abscess caused by Klebsiella pneumoniae: single-stage operation with decompressive corpectomy and autologous bone fusion. Kor J Spine.

[11] Hanifah J, Joehaimey J, Yusof MI (2017). A good short-term outcome in delayed decompression of cauda equina syndrome in Klebsiella pneumoniae spinal epidural abscess: a case report. Malays Orthop J.

